# Study of Osteoarthritis Treatment with Anti-Inflammatory Drugs: Cyclooxygenase-2 Inhibitor and Steroids

**DOI:** 10.1155/2015/595273

**Published:** 2015-04-27

**Authors:** Hongsik Cho, Andrew Walker, Jeb Williams, Karen A. Hasty

**Affiliations:** ^1^Department of Orthopaedic Surgery and Biomedical Engineering, University of Tennessee Health Science Center and Campbell Clinic, Memphis, TN, USA; ^2^Veterans Affairs Medical Center, Memphis, TN, USA

## Abstract

Patients with osteoarthritis (OA), a condition characterized by cartilage degradation, are often treated with steroids, nonsteroidal anti-inflammatory drugs (NSAIDs), and cyclooxygenase-2 (COX-2) selective NSAIDs. Due to their inhibition of the inflammatory cascade, the drugs affect the balance of matrix metalloproteinases (MMPs) and inflammatory cytokines, resulting in preservation of extracellular matrix (ECM). To compare the effects of these treatments on chondrocyte metabolism, TNF-*α* was incubated with cultured chondrocytes to mimic a proinflammatory environment with increasing production of MMP-1 and prostaglandin E2 (PGE2). The chondrocytes were then treated with either a steroid (prednisone), a nonspecific COX inhibitor NSAID (piroxicam), or a COX-2 selective NSAID (celecoxib). Both prednisone and celecoxib decreased MMP-1 and PGE-2 production while the nonspecific piroxicam decreased only the latter. Both prednisone and celecoxib decreased gene expression of MMP-1 and increased expression of aggrecan. Increased gene expression of type II collagen was also noted with celecoxib. The nonspecific piroxicam did not show these effects. The efficacy of celecoxib *in vivo* was investigated using a posttraumatic OA (PTOA) mouse model. *In vivo*, celecoxib increases aggrecan synthesis and suppresses MMP-1. In conclusion, this study demonstrates that celecoxib and steroids exert similar effects on MMP-1 and PGE2 production *in vitro* and that celecoxib may demonstrate beneficial effects on anabolic metabolism *in vivo*.

## 1. Introduction

Osteoarthritis (OA) is the leading cause of pain and disability in older individuals in the US. Currently, there is no cure for OA, and the standards of treatment are primarily limited to pain management, steroids and other anti-inflammatory drugs, physical therapy, and eventual joint replacement [1]. Posttraumatic OA (PTOA) occurs after joint, ligament, or bone injury or surgery. In all types of OA, mechanical stress and overuse result in stimulation of proinflammatory cytokines like TNF-*α* (tumor necrosis factor) and matrix metalloproteinases (MMPs) [2–4]. These MMPs (especially 1 and 13) degrade type II collagen (CII) resulting in focal lesions in the articular surface [5–7]. In this mechanism, TNF-*α* plays a key role in the degradation process by stimulating expression and release of proteases, such as collagenases, aggrecanases, and MMPs, which degrade collagen and aggrecan. Additionally, these proinflammatory cytokines stimulate synthesis and release of nitric oxide (NO) and prostaglandin E2 (PGE2) [8].

The anti-inflammatory effects of NSAIDs are mainly due to their ability to inhibit cyclooxygenase (COX), impairing production of prostaglandins, which are important mediators of both pain and the inflammatory response. COX enzymes metabolize arachidonic acid, forming prostaglandin H2, which is subsequently metabolized by prostaglandin E synthase into prostaglandin E2 (PGE2) [9, 10]. There are two isoforms of the COX enzyme: COX-1, found in most tissues and constitutively expressed in normal cells, and COX-2, which is not expressed in healthy tissue but is induced by various catabolic mediators, such as cytokines, growth factors, and mechanical stress [11]. Beneficial effects of NSAIDs on inflammation are mediated by COX-2 inhibition, whereas unwanted gastrointestinal effects are caused by primarily inhibition of COX-1 [12]. This data initially popularized the use of selective COX-2 inhibitors. Celecoxib (SC-58635; 4-[5-(4-methylphenyl)-3-(trifluoromethyl)-1H-pyrazol-1-yl]benzenesulfon-amide), the only FDA approved COX-2 inhibitor, has been used in the treatment of OA [13, 14].

Previous studies show that nonsteroidal anti-inflammatory drugs (NSAIDs) may have beneficial effects on cartilage damage through their inhibition of PGE2 production [15, 16]. PGE2, derived from the activity of IL-1*β* and TNF-*α*, results in decreased proteoglycan content in cartilage explants. [17]. Cartilage from joint replacement surgery patients treated with celecoxib, a COX-2 selective inhibitor, showed a higher rate of proteoglycan synthesis and a better retention of the newly formed proteoglycans, effects which preserve the articular surface and delay OA. Nonspecific COX inhibitors did not demonstrate these findings but instead showed a tendency towards a lower synthesis rate of proteoglycans [18]. A better understanding of mechanisms and the timing of significant biologic events in the development of OA and PTOA will allow investigators to determine optimal timing for biologic interventions in the future [4].

Current minimalist therapies are only palliative and little is done to prevent cartilage damage. Because treatment does not occur until after painful symptoms present, minimalist therapies (rest, ice, short term NSAIDS, elastic support) for traumatic or sport injuries whether or not they are surgically treated may be missing an opportunity to prevent or slow OA development with early pharmacological intervention.

In previous studies, we have accomplished early OA detection using a fluorescent selectively binding monoclonal antibody (Mab) which binds exposed CII in the articular cartilage. Binding is measured with a near-infrared imaging system. The amount of binding is observed to be proportional to the extent of the damage to the cartilage [19].

In this study, we subject chondrocytes* in vitro* to a proinflammatory dose of TNF-*α* (thus mimicking an activated chondrocyte) and subsequently compare the efficacy of a steroid (prednisone), a COX-2 selective inhibitor (celecoxib), and a nonselective COX inhibitor (piroxicam) for reducing catabolic MMP and PGE2 production and stimulating anabolic CII and aggrecan production [20]. We have also investigated the effect of the COX-2 inhibitor on the progression of PTOA* in vivo*. This is accomplished using a mechanically loaded PTOA mouse model and this lab's previously studied monoclonal antibody damage detection system. Mechanical loading induces inflammatory change through the nuclear factor-*κ*B (NF-*κ*B) pathway, thus initiating proinflammatory action [21]. Use of a mechanically loaded PTOA model will allow future longitudinal studies of drug efficacy without the need for sacrificing animals during the study. This preliminary study with the PTOA model establishes a noninvasive, easily reproducible method for initiating inflammatory PTOA changes in murine joints. Fluorescently tagged antibodies bind to exposed articular cartilage [19]. This binding is measured as radiant efficiency using IVIS scanning of the intact murine joint [19]. Modifications to the disease state over the course of treatment with drugs such as celecoxib can be continuously followed. Results from this preliminary study will guide future research in this direction.

## 2. Methods

### 2.1. Cell Culture

The primary chondrocytes used in this investigation were aseptically harvested from the articular cartilage of the femoral condyles of domestic pigs ranging from 25 to 35 kg. Articular cartilage was removed from the condyles in thin sections by cutting just beneath the surface in a direction that paralleled the natural curvature of the condyles. All tissues were taken from the knees of healthy pigs freshly sacrificed for other experiments according to approved protocols and experimental procedures of the University of Tennessee Health Science Center.

The chondrocytes were isolated by 1-2-hour digestion at 37°C in 0.05% Pronase (Boehringer Mannheim, Mannheim, Germany), followed by overnight digestion in at 37°C in 0.2% collagenase (Worthington Biologicals, Lakewood, NJ) using modified F-12K medium (Invitrogen, Grand Island, NY) with 5% fetal calf serum (FCS, Atlanta Biologicals, GA). The cells were then plated at 15,000 cells/cm^2^. Cells were cultured at 37°C in a humidified atmosphere of 5% CO_2_ and in F-12K medium supplemented with 10% FCS, streptomycin (50 *μ*g/mL; Invitrogen), penicillin G (50 IU/mL; Invitrogen), L-glutamine (2 mM; Invitrogen), and ascorbic acid (50 *μ*g/mL; Invitrogen). The medium was changed every other day until the cells were confluent.

### 2.2. Drug Preparation and Treatment

Piroxicam, celecoxib, and prednisone were purchased in powdered form from Sigma-Aldrich (St. Louis, MO). Drugs were prepared for three treatment groups: celecoxib (10 *μ*M), piroxicam (5 *μ*M), and prednisone (5 *μ*M). All drugs had minimal solubility in water; thus 10 *μ*L of DMSO was used to fully dissolve 1 mg of each drug. The drug/DMSO mixture was then diluted to the desired treatment molarity with serum free F12K media containing 5 mL penicillin/streptomycin and ascorbic acid (50 *μ*g/mL). To activate chondrocytes and stimulate an inflammatory response, all groups were dosed with 5 ng/mL of TNF-*α*. After 24 hours, the supernatant media were removed for Western blot analysis for MMP-1 and ELISA for PGE2.

### 2.3. PTOA Mouse Model and* In Vivo* Drug Treatment

Twenty-four C57BL/6 male mice (age of 10 weeks at time of mechanical loading; body weight of ~20 g with <10% variance) were obtained from Jackson Laboratory (Maine, USA). Mice were randomly divided into two test groups: a control to which a mechanical load was applied to the left knee without drug treatment and a mechanically loaded group treated with celecoxib. All procedures, in this study, were performed according to approved protocols and experimental procedures of IACUC at the University of Tennessee Health Science Center. In order to prepare for mechanical loading, the mice were placed in an anesthetic induction chamber and anesthetized continuously with 2% isoflurane. The left leg of each mouse was positioned into a custom made loading apparatus within the calibrated ElectroForce 3200 (Bose Corp., MN, USA) biomaterials test instrument. The distal femur rested in the upper cup and the dorsiflexed ankle was inserted into the bottom cup of the apparatus (Figure 7, schematic diagram). In order to execute the loading protocol, the left knee joint of each mouse received 40 cycles of compressive loading at 9 N, three times weekly for two weeks. These methods were adapted from Poulet's protocol [22]. The mice were allowed to have normal activity in between and after load applications. The loading data was collected for each mouse using WinTest software (Bose Corp., MN, USA). All mice were treated with 5 weeks of either celecoxib or saline with 0.1% DMSO by daily gavaging beginning on day 1 of mechanical loading. Celecoxib was dissolved in saline with 0.1% DMSO and administered at a dosage of 10 mg/kg/day (0.268 mg in 100 *μ*L) per Cottrell's previous study [23]. Each dose was equal and administered according to the same therapeutic regimen based on the average initial weight of all experimental mice. The mice were allowed to ingest oral food and water ad libitum.

### 2.4. Cell Viability

For counting and general microscopic observations, isolated chondrocytes were stained with 0.4% trypan blue dye and counted under light microscopy using a 0.1 mm deep hemocytometer (Reichert, Buffalo, NY).

### 2.5. PGE2 Enzyme-Linked Immunosorbent Assay (ELISA)

After 24 hours of treatment, supernatants were collected from each well and analyzed for PGE2 concentration with an Enzyme Immunoassay kit (Item number 514010, Cayman Chemical Co., Inc., Ann Arbor, MI). Finally, the microtiter plate was read at a wavelength of 405 nm using a plate reader (SPECTRAmaxTM, Molecular Devices Corp., CA).

### 2.6. Western Blot for MMP-1

After 24 hours in culture, the supernatants were collected and proteins isolated. Protein concentrations were normalized by cell number and then mixed with Laemmli sample buffer, separated by SDS/PAGE, and electrophoretically transferred to PVDF membranes (GE Healthcare, PA). Blots were blocked for 1 h in Tris-buffered saline with 5% milk and incubated overnight at 4°C with primary antibodies to MMP-1. Membranes were washed in Tris-buffered saline, incubated with HRP conjugated secondary antibodies, and washed. Immunoreactive bands were visualized by incubation with ECF substrate (Amersham, PA).

### 2.7. Optical and Histopathological Analysis

Cartilage damage in early OA was quantitated using a fluorescent monoclonal antibody that binds exposed CII (MabCII) in the articular cartilage. Binding of the antibody to the damaged cartilage* in vivo* is measured with an optical imaging system. We have shown that the amount of binding is observed to be proportional to the extent of the damage to the cartilage [19]. To determine the amount of cartilage damage* in vivo*, mice were injected retroorbitally with 80 *μ*L of solution containing near-infrared fluorescent dye (NIF) conjugated to type II collagen antibody using a XenoLight CF680 Labeling kit (Caliper Life Science, MA). After 24 hours, the mice were anesthetized, depilated, and scanned using the* in vivo* imaging system (IVIS Lumina XR System, Perkin Elmer, Hopkinton, MA) with a mid-high range filter set (excitation 675 nm, emission 720 nm). The fluorescence remaining in each knee joint was quantified using Living Image 4.0 software to calculate the flux radiating omnidirectionally from the region of interest (ROI) and graphed as radiant efficiency (photons/sec/cm^2^/str)/(*μ*W/cm^2^). To yield a standardized ROI for measurement of the knee fluorescence, the same area of capture was used for each mouse. Fluorescence from a null or background capture area (consisting of muscle and skin tissue) was measured and subtracted from each articular reading [19]. After IVIS imaging, the mice were sacrificed, and their knees were dissected and the femoral tibial and patella portions were reimaged separately by IVIS. The knees were dissected to get cartilage tissues and then were put into RNAlator in order to isolate RNA for rtPCR. Also, some of knees were then fixed in 10% formalin solution for histopathological analysis and decalcified with Decalcifying Solution (Thermo Scientific, MA) before embedding in paraffin. Twenty histological sections, each taken 200 *μ*m apart, were analyzed for arthritic joint damage across the entire joint. The sections were stained with H&E.

### 2.8. Quantitative Real-Time Reverse Transcription-Polymerase Chain Reaction (RT-PCR)

RNA was extracted from the cells with TRIzol reagent (Invitrogen Life Technologies, Carlsbad, CA) according to the manufacturer's protocol. To measure target gene expression, we used an ABI Prism 7900 Sequence Detection System (Applied Biosystems, Foster City, CA) for RT-PCR with custom designed primers and fluorescently labeled oligonucleotide probes specific for porcine CII (Ss03373343_g1), aggrecan (Ss03373377_m1), MMP-1 (Ss04245659_m1), MMP-13 (Ss03373279_m1), and the housekeeping gene, *β*-actin (Ss03375629_u1) for* in vitro* test.* In vivo*, we used the probe CII (Mm00491889_m1), aggrecan (Mm00545794_m1), MMP-13 (Mm00439491_m1), and *β*-actin (Mm00607939_s1) but not MMP-1 as this gene is not highly expressed in mice. All primer and probe sets were purchased from Applied Biosystems. According to the manufacturer's protocol, the cycle threshold (Ct) values were measured and the relative transcription levels were calculated. The data was plotted as a relevant expression calculated as 2^−ΔΔCt^, where the cycle threshold is the beginning of the logarithmic amplification of the probe set, and ΔCt is the difference of the target gene Ct subtracted from the housekeeping gene Ct. Data was then calculated as 2 (the increase in probe signal generated with each cycle) to the negative exponential value of ΔCt and plotted as a relative change to either controls or the TNF-*α* stimulated group [24–26].

### 2.9. Statistics

All experiments were performed independently at least three times. Microsoft Excel with Student's *t*-test and analysis of variance were used to determine statistical significance. A *P* value of less than 0.05 was considered statistically significant. Also, a one-way ANOVA test was performed for analysis of cell viability.

## 3. Results

In order to show the efficacy of the COX-2 inhibitor in reducing cartilage damage, a catabolic state was induced* in vitro* with articular chondrocytes. This was accomplished with the introduction of the cytokine TNF-*α* at an optimized concentration of 5 ng/mL, determined experimentally by RT-PCR for MMP-1 gene expression as shown in Supplemental Figure 1 in Supplementary Material available online at http://dx.doi.org/10.1155/2015/595273. These results were previously reported [27].

**Figure 1:**
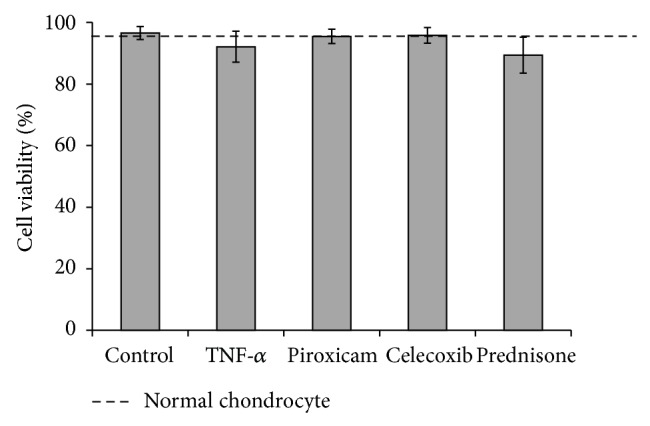
Cell viability. Chondrocytes viabilities were measured using trypan blue method after 24 hours of treatment with the experimental drugs. As the figure indicates, none of the drugs had an effect on cell viability as compared to the control group. The dotted line denotes normal chondrocytes untreated with TNF-*α* or drugs.

Pig chondrocytes showed no change in cell viability after treatment with the various drugs, nor was there any difference between the cells stimulated with only TNF-*α* (5 ng/mL) compared to cells receiving different drugs. There were no statistically significant differences in cell viability between groups, and the average cell viability among the groups was greater than 90% (Figure 1).

**Figure 2:**
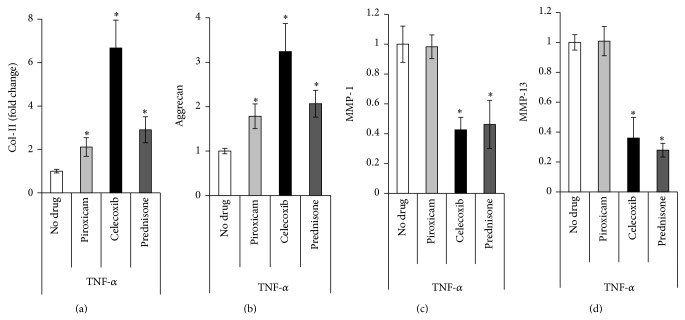
Gene expression* in vitro* after treatment with TNF-*α* with or without additional drugs. Changes in CII (a), aggrecan (b), and MMP-13 (c) gene expression after treatment with TNF-*α* (5 ng/mL) with or without drug treatment. Gene expression after treatment with TNF-*α* only is set as the base case (equal to 1). Gene expression of the drug treated chondrocytes is relative to the “no drug” case which contains TNF-*α* but no drug treatment. (∗ indicates *P* < 0.05).

Figure 2 shows gene expression of CII, aggrecan, and MMP-13 after treatment with the various drugs and TNF-*α*. All drug treatments significantly increased anabolic activity as evidenced by CII and aggrecan expression. Treatment with celecoxib resulted in a 6-fold increase in CII expression over the control. Piroxicam and prednisone increased CII expression by 2- and 3-fold, respectively. Celecoxib increased aggrecan expression by 3.2-fold, whereas piroxicam and prednisone increased aggrecan by 1.8- and 2-fold, respectively. Treatments with either celecoxib or prednisone were shown to decrease expression of the proteinase MMP-13 in a statistically equivalent manner, about 70% below the TNF stimulated level. Piroxicam had no effect on MMP-13 expression.

**Figure 3:**
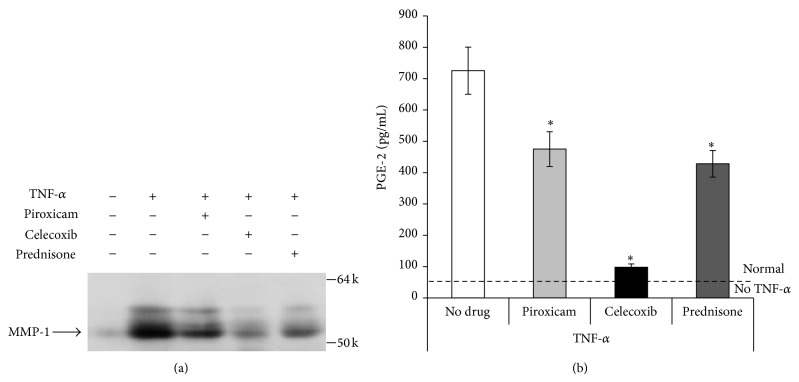
Protein production assays for MMP-1 and PGE2* in vitro* after treatment with TNF-*α* with or without additional drugs. MMP-1 and PGE2 after treatment with TNF-*α* (5 ng/mL) with or without drug treatment. (a) shows that TNF-*α* induced MMP-1 production (compare columns 1 and 2). All drug/TNF-*α* combinations result in less MMP-1 production, with celecoxib reducing production of MMP-1 most dramatically. (b) shows that TNF-*α* alone induces PGE2 production. All drugs significantly reduce PGE-2 production, celecoxib being the most effective. The dotted line represents PGE-2 production with no TNF-*α* and no drug treatment.

Western blot analyses show the effect of pharmacologic treatment on MMP-1 production (Figure 3(a)) by chondrocytes stimulated with TNF-*α in vitro*. The proinflammatory cytokine TNF-*α* increases both the gene expression of MMP-1 (Supplemental Figure 1) and concentration of the MMP-1 protein as measured by Western blot. Similarly, the PGE2 ELISA shows the effects that TNF-*α* and pharmacologic treatments have on PGE2 production by porcine chondrocytes (Figure 3(b)). Both assays showed comparable trends. Chondrocytes treated with TNF-*α* alone showed increased production of both MMP-1 and PGE2. However, when the TNF-*α* was combined with pharmacologic treatment, the production of MMP-1 and PGE2 was reduced. Celecoxib, the COX-2 selective inhibitor, proved to be the most effective at decreasing the production of the inflammatory cytokines, decreasing PGE2 concentration by 90%. Piroxicam, the nonselective COX inhibitor, and prednisone, the steroid, had a substantial effect, but they were less effective than celecoxib.

**Figure 4:**
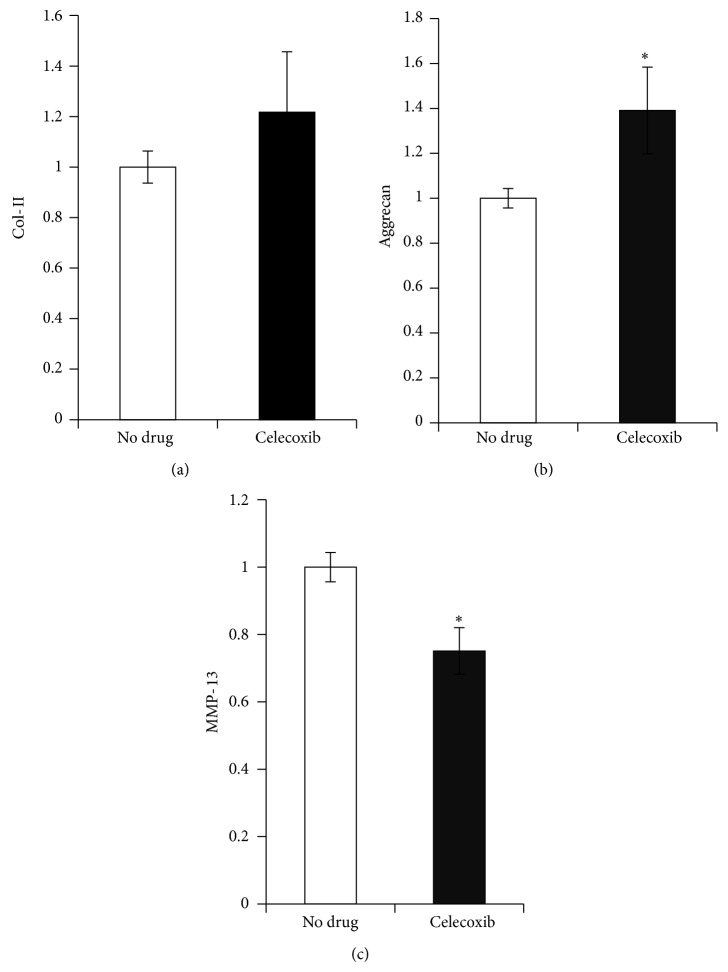
*In vivo* gene expression of cartilage from mechanically loaded mouse knees. Cartilage of mechanically loaded knees after treatment with celecoxib (10 mg/kg) by gavage for 5 weeks demonstrated increased gene expression of anabolic markers CII and aggrecan as compared to a mechanically loaded knees of mice treated with saline alone “no drug” (a). The increase in aggrecan gene expression reached statistical significance (b). Treatment with celecoxib significantly decreased MMP-13 gene expression as compared to the “no drug” control (c) (*n* = 6 for each treatment group). The “no drug” group has been treated with TNF-*α*, but not celecoxib.

*In vitro* data shows celecoxib to be an effective agent both increasing anabolic activity of chondrocytes and decreasing protease activity. To more closely approximate a clinical scenario, celecoxib was administered to a murine PTOA model in mice where a knee joint was compressively loaded in a repetitive manner. These mice were subjected to mechanical loading and simultaneously treated for 5 weeks with either celecoxib or saline. At the end of this time the expression of CII, aggrecan, and MMP-13 was measured in cartilage samples taken from the mechanically loaded knee joint. Celecoxib treatment significantly increased production of aggrecan and decreased MMP-13 over that seen in mechanically loaded mice receiving no treatment. CII production was not significantly increased by celecoxib treatment but tended to trend upward (Figure 4).

**Figure 5:**
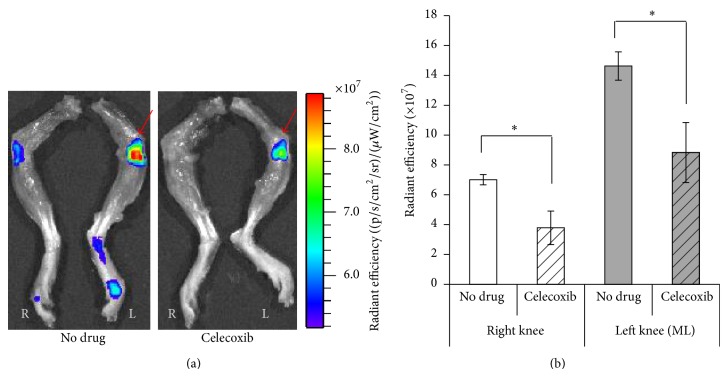
Optical imaging of fluorescently labelled MabCII antibody in PTOA model mice with and without treatment with celecoxib. IVIS scanning shows fluorescent CII targeted antibody binding to the damaged cartilage surface. (a) shows the antibody binding in greater quantity to the loaded left knee in both the drug treated and nondrug treated cases. Antibody binding to the loaded knee of the celecoxib treated group is lower than the loaded knee of the nondrug treated group. The red arrow indicates a mechanically loaded knee. (b) quantifies this binding by measuring fluoresce intensity and calculating radiant efficiency. Results are parallel (a) (*n* = 6 for each treatment group).

**Figure 6:**
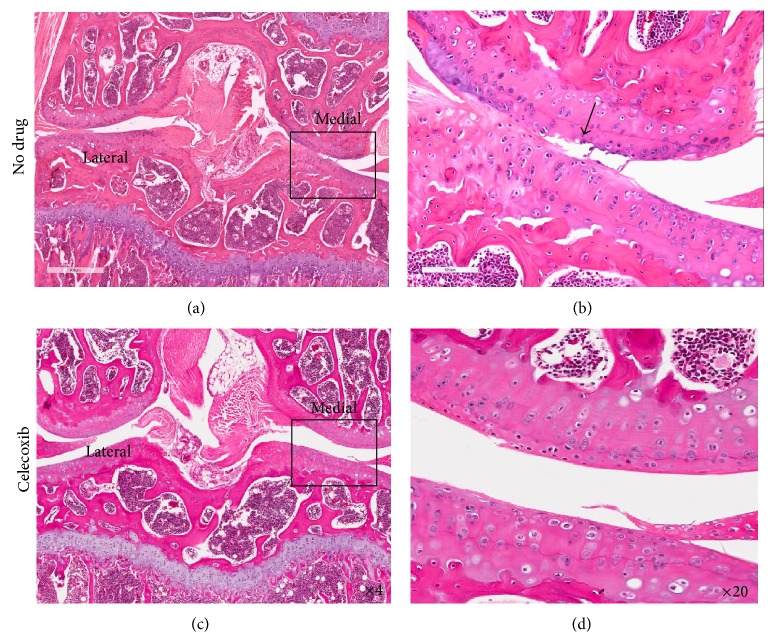
Histopathology of mechanically loaded knee joints with and without treatment with celecoxib. This figure shows H&E stained coronal sections of loaded mouse knees. Twenty histological sections, each taken 200 *μ*m apart, were analyzed for arthritic joint damage across the entire joint. Sections at the same depth relative to the patella were compared. (a) and (b) are from the mechanically loaded knees of mice receiving no drug treatment (only saline). (c) and (d) are from the mechanically loaded knees of mice treated with celecoxib. In both cases, the lateral tibial and femoral plateaus have minimal damage. The medial plateaus sustained more damage, and thus higher magnification ((b) and (d)) compares treated (d) and untreated medial plateaus (b).

**Figure 7:**
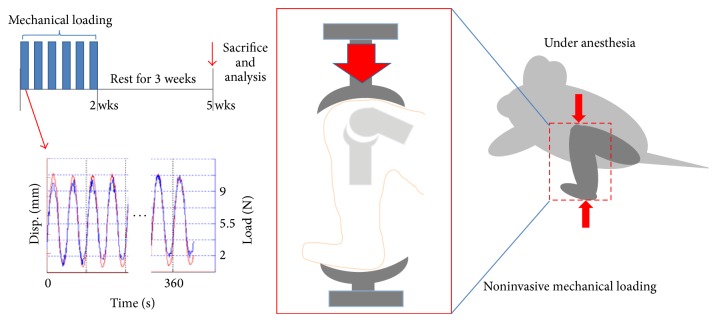
Mechanically loaded PTOA mouse model schematic diagram. This figure graphically represents the cycle of loading (6 cycles) over the course of 2 weeks followed by 3 weeks of rest. The figure also demonstrates the position of the mouse knee in the loading apparatus and the force versus time diagram.

In previous studies, IVIS scanning has been used to quantify the degree of cartilage damage due to PTOA [28]. In this study, we adapt this method to show how treatment with celecoxib alters damage severity. This effect is further confirmed by histological evaluation.  Figure 5 shows florescence intensity in both celecoxib and saline treated PTOA mice. Due to the selectively binding nature of MabCII-NIF, this intensity (ROI) directly correlates with cartilage damage. The MAbCII-NIF showed selective binding to the damaged cartilage in the mechanically loaded left knee (Figure 5(a)). The loaded left knee of the celecoxib treated mouse shows a lower signal intensity and ROI (Figure 5(b)) as compared to the loaded left knee of the saline treated mouse, suggesting less damage to the articular surface after treatment with celecoxib. These results correlate with histology of the loaded joints. The loaded joint treated with saline (Figures 6(a) and 6(b)) shows extensive damage to the articular surface. The loaded knee of celecoxib treated mouse (Figures 6(c) and 6(d)) shows a more intact articular surface. The superficial articular layer in the celecoxib-treated knees demonstrates a more linear, intact structure than that of the saline treated knees. Chondrocytes in the deeper zone of the celecoxib-treated knee (Figure 6(d)) do not show the increased cell proliferation within the isogenous groups seen in the nontreated mechanically loaded knee (Figure 6(b)).

## 4. Discussion

In OA, chondrocyte homeostasis becomes imbalanced for synthesis and degradation of the extracellular matrix, resulting in progressive disruption of articular cartilage. TNF-*α* and other cytokines may play key roles in the destructive process by triggering release of proteases, such as matrix metalloproteinases (MMPs), which degrade collagen. As shown in this study, we used TNF-*α* (5 ng/mL) to stimulate cultured chondrocytes and to mimic a catabolic environment* in vitro* [27]. This proinflammatory cytokine stimulated synthesis and release of PGE2 [8]. The function of PGE2 in OA is not entirely clear as it has both catabolic and anabolic effects on cartilage [15, 16]. In OA, however, chondrocyte expression of COX-2 increases, thereby increasing PGE2 concentration to nano- to micromolar concentrations [29, 30]. Hardy et al. demonstrate that both COX-2 and PGE2 effect proteoglycan production in OA chondrocytes in a concentration dependent manner; namely, high concentrations of both reduce proteoglycan synthesis [31]. Several studies suggest that at nanomolar concentrations PGE2 exerts a catabolic influence. Specifically, they have found that, in OA cultures treated with celecoxib, the direct addition of PGE2 negates the beneficial effect of celecoxib on MMP, CII, and aggrecan levels [30, 31]. Additionally, research by Attur et al. shows that exogenous PGE2 increases MMP-13 expression which in turn increases collagen breakdown and reduces proteoglycan synthesis [30]. In a separate study this group of researchers demonstrated that treatment with celecoxib results in decreased MMP-1 expression. In our study, PGE2, MMP-13, and MMP-1 expressions are all observed to decrease after celecoxib treatment. NSAIDs could potentially affect cartilage through inhibition of PGE2 production [14, 31]. Other studies show that NSAIDs function to prevent cartilage damage. Cartilage from patients treated before joint replacement surgery with the COX-2 inhibitor celecoxib showed a higher rate of proteoglycan synthesis and a better retention of the newly formed proteoglycans. In contrast, nonspecific COX inhibitors showed a tendency towards a decreased rate of proteoglycan synthesis [18]. The* in vitro* concentration of celecoxib used in these experiments was taken from Mastbergen et al. who demonstrated that proteoglycan turnover was greatest at 10 *μ*M.* In vivo* dosing of celecoxib (10 mg/kg) was adapted from Cottrell and O'Connor's dosage for mice [23]. Our dose of piroxicam is the maximum dose possible without yielding significant effects on cell division and viability as demonstrated by Chang et al. [32]. The* in vitro* data obtained in this study shows that chondrocytes treated with celecoxib produce fewer arthritis-associated mediators such as PGE2 and MMP-1 than chondrocytes treated with piroxicam or prednisone and more anabolic indicators, namely, CII and aggrecan.

In our study and others, nonselective COX inhibitors, represented in our experiment by piroxicam, have been shown to have minimal beneficial effect on proteoglycan turnover and repair [33, 34]. This difference in NSAID effect supports COX-2 involvement in catabolic activity regulation in cartilage, whereas COX-1 activity may have a more constitutive role in chondrocytes [14, 35]. We chose to investigate the COX-2 inhibitor* in vivo* with the hypothesis that preserving the COX-1-mediated constitutive role, while inhibiting the induced COX-2 activity, would optimize OA treatment and prevention. Furthermore, few studies have described the effects of celecoxib on cartilage destruction* in vivo* [14, 36–39]. In* ex vivo* studies, 4 weeks of celecoxib treatment is shown to have beneficial effects on proteoglycan synthesis rates and proteoglycan retention, though no differences in the histopathological Mankin score were observed [38]. Studies of this kind are limited by sample size and duration and the lack of a reproducible animal model for OA and drug monitoring. Other animal models of PTOA require direct surgical manipulation and lead to aggressive and rapid joint destruction. Our studies utilize a PTOA animal model that requires no invasive intervention. The mechanical loading technique is easily reproducible and provides an alternate nonsurgical means of replicating the pathophysiology of PTOA.

Trauma which gives rise to osteoarthritic change is accompanied by injury of the adjacent soft tissues resulting in an increase of proinflammatory cytokines such as TNF-*α* in the joint space. As such, the combined action of trauma-induced and cytokine-induced processes in cartilage characterizes the early development of PTOA (Lewis et al.). Coupled with a noninvasive method of monitoring OA damage, this model could help researchers continuously monitor OA progression and the efficacy of drugs over the disease course. The noninvasive monitoring technique described in this experiment uses a fluorescently labeled monoclonal antibody targeted to CII (MabCII-NIF) and IVIS scanning. Previous studies by this lab have shown this method to be effective at identifying early stages of OA corresponding to histopathological Mankin scores less than 3 [19, 28]. Early changes of PTOA occur within 24 hours of joint injury. These changes include chondrocyte apoptosis and a significant surge in proinflammatory cytokines, nitric oxide, free radicals, and MMPs resulting in cartilage matrix damage [4, 40–44]. Therapies to target this phase need to be given early in disease when both clinical symptoms and histopathological Mankin scores are low [4].

As shown at our animal study the celecoxib reduced MMP expression and delayed the progress of arthritic damage in PTOA. However, for long-term therapy with this drug, the side effect of cardiac risk must be considered [45, 46]. The targeted antibody used in this study selectively binds to tissue demonstrating early OA changes and can be conjugated to a drug-encapsulating nanoscale liposome (nanosome) [19]. Delivery of therapeutic agents such as celecoxib with this nanosome may bring timely effective treatment to the site of joint damage, maximizing local drug delivery while minimizing systemic side effects. The mechanically loaded PTOA mouse model combined with IVIS scanning provides a means to monitor drug effects using this treatment method longitudinally without animal sacrifice [28]. The combination of early detection with aggressive, targeted pharmacologic treatment could have an enormous impact on the treatment and prevention of OA while reducing the side effect burden of common effective treatments.

## Supplementary Material

S. Figure 1. The effect of TNF-a concentration on MMP-1 and MMP-13 gene expression in pig chondrocytes.
S. Figure 2. Effect of COX-2 inhibitor on MMP-1 and MMP-13 gene expression.
S. Figure 3. Cell Viability in 0.1% DMSO.

## References

[1] Zhang W., Moskowitz R. W., Nuki G. (2007). OARSI recommendations for the management of hip and knee osteoarthritis, part I: critical appraisal of existing treatment guidelines and systematic review of current research evidence. *Osteoarthritis and Cartilage*.

[2] Guilak F., Fermor B., Keefe F. J. (2004). The role of biomechanics and inflammation in cartilage injury and repair. *Clinical Orthopaedics and Related Research*.

[3] Ko F. C., Dragomir C., Plumb D. A. (2013). In vivo cyclic compression causes cartilage degeneration and subchondral bone changes in mouse tibiae. *Arthritis & Rheumatism*.

[4] Olson S. A., Horne P., Furman B. (2014). The role of cytokines in posttraumatic arthritis. *Journal of the American Academy of Orthopaedic Surgeons*.

[5] Mitchell P. G., Magna H. A., Reeves L. M. (1996). Cloning, expression, and type II collagenolytic activity of matrix metalloproteinase-13 from human osteoarthritic cartilage. *Journal of Clinical Investigation*.

[6] Smith G. N. (2006). The role of collagenolytic matrix metalloproteinases in the loss of articular cartilage in osteoarthritis. *Frontiers in Bioscience*.

[7] Martel-Pelletier J., Boileau C., Pelletier J.-P., Roughley P. J. (2008). Cartilage in normal and osteoarthritis conditions. *Best Practice & Research: Clinical Rheumatology*.

[8] Goldring M. B. (2000). Osteoarthritis and cartilage: the role of cytokines. *Current Rheumatology Reports*.

[9] Vane J. R. (1971). Inhibition of prostaglandin synthesis as a mechanism of action for aspirin-like drugs. *Nature: New Biology*.

[10] Crofford L. J., Lipsky P. E., Brooks P., Abramson S. B., Simon L. S., van de Putte L. B. A. (2000). Basic biology and clinical application of specific cyclooxygenase-2 inhibitors. *Arthritis & Rheumatology*.

[11] Martel-Pelletier J., Pelletier J. P., Fahmi H. (2003). Cyclooxygenase-2 and prostaglandins in articular tissues. *Seminars in Arthritis and Rheumatism*.

[12] Hawkey C. J. (1999). COX-2 inhibitors. *The Lancet*.

[13] Bingham C. O. (2002). Development and clinical application of COX-2-selective inhibitors for the treatment of osteoarthritis and rheumatoid arthritis. *Cleveland Clinic Journal of Medicine*.

[14] Zweers M. C., de Boer T. N., van Roon J., Bijlsma J. W. J., Lafeber F. P. J. G., Mastbergen S. C. (2011). Celecoxib: considerations regarding its potential disease-modifying properties in osteoarthritis. *Arthritis Research & Therapy*.

[15] Tchetina E. V., di Battista J. A., Zukor D. J., Antoniou J., Poole A. R. (2007). Prostaglandin PGE2 at very low concentrations suppresses collagen cleavage in cultured human osteoarthritic articular cartilage: this involves a decrease in expression of proinflammatory genes, collagenases and COL10A1, a gene linked to chondrocyte hypertrophy. *Arthritis Research & Therapy*.

[16] Attur M., Al-Mussawir H. E., Patel J. (2008). Prostaglandin E2 exerts catabolic effects in osteoarthritis cartilage: evidence for signaling via the EP4 receptor. *The Journal of Immunology*.

[17] Mastbergen S. C., Bijlsma J. W. J., Lafeber F. P. J. G. (2008). Synthesis and release of human cartilage matrix proteoglycans are differently regulated by nitric oxide and prostaglandin-E2. *Annals of the Rheumatic Diseases*.

[18] Mastbergen S. C., Lafeber F. P. J. G., Bijlsma J. W. J. (2002). Selective COX-2 inhibition prevents proinflammatory cytokine-induced cartilage damage. *Rheumatology*.

[19] Cho H., Stuart J. M., Magid R. (2014). Theranostic immunoliposomes for osteoarthritis. *Nanomedicine: Nanotechnology, Biology, and Medicine*.

[20] Tsutsumi R., Ito H., Hiramitsu T. (2008). Celecoxib inhibits production of MMP and NO via downregulation of NF-kappaB and JNK in a PGE2 independent manner in human articular chondrocytes. *Rheumatology International*.

[21] Deschner J., Hofman C. R., Piesco N. P., Agarwal S. (2003). Signal transduction by mechanical strain in chondrocytes. *Current Opinion in Clinical Nutrition and Metabolic Care*.

[22] Poulet B., Hamilton R. W., Shefelbine S., Pitsillides A. A. (2011). Characterizing a novel and adjustable noninvasive murine joint loading model. *Arthritis & Rheumatism*.

[23] Cottrell J., O'Connor J. P. (2010). Effect of non-steroidal anti-inflammatory drugs on bone healing. *Pharmaceuticals*.

[24] Overbergh L., Valckx D., Waer M., Mathieu C. (1999). Quantification of murine cytokine mRNAs using real time quantitative reverse transcriptase PCR. *Cytokine*.

[25] Giulietti A., Overbergh L., Valckx D., Decallonne B., Bouillon R., Mathieu C. (2001). An overview of real-time quantitative PCR: applications to quantify cytokine gene expression. *Methods*.

[26] Huang J., Ballou L. R., Hasty K. A. (2007). Cyclic equibiaxial tensile strain induces both anabolic and catabolic responses in articular chondrocytes. *Gene*.

[27] Cho H., Lee S., Park S.-H., Huang J., Hasty K. A., Kim S.-J. (2013). Synergistic effect of combined growth factors in porcine intervertebral disc degeneration. *Connective Tissue Research*.

[28] Cho H., Pinkhassik E., David V., Stuart J. M., Hasty K. A. (2015). Detection of early cartilage damage using targeted nanosomes in a post-traumatic osteoarthritis mouse model. *Nanomedicine: Nanotechnology, Biology and Medicine*.

[29] White B., Schmidt M., Murphy C. (2000). Activated protein C inhibits lipopolysaccharide-induced nuclear translocation of nuclear factor *κ*B (NF-*κ*B) and tumour necrosis factor *α* (TNF-*α*) production in the THP-1 monocytic cell line. *British Journal of Haematology*.

[30] Attur M., Al-Mussawir H. E., Patel J. (2009). Prostaglandin E_2_ exerts catabolic effects in osteoarthritis cartilage: evidence for signaling via the EP4 receptor. *The Journal of Immunology*.

[31] Hardy M. M., Seibert K., Manning P. T. (2002). Cyclooxygenase 2-dependent prostaglandin E2 modulates cartilage proteoglycan degradation in human osteoarthritis explants. *Arthritis and Rheumatism*.

[32] Chang J.-K., Wang G.-J., Tsai S.-T., Ho M.-L. (2005). Nonsteroidal anti-inflammatory drug effects on osteoblastic cell cycle, cytotoxicity, and cell death. *Connective Tissue Research*.

[33] El Hajjaji H., Marcelis A., Devogelaer J.-P., Manicourt D.-H. (2003). Celecoxib has a positive effect on the overall metabolism of hyaluronan and proteoglycans in human osteoarthritic cartilage. *The Journal of Rheumatology*.

[34] Mastbergen S. C., Jansen N. W. D., Bijlsma J. W. J., Lafeber F. P. J. G. (2006). Differential direct effects of cyclo-oxygenase-1/2 inhibition on proteoglycan turnover of human osteoarthritic cartilage: an in vitro study. *Arthritis Research & Therapy*.

[35] Nakamura H., Masuko K., Yudoh K., Kato T., Nishioka K. (2007). Effects of celecoxib on human chondrocytes—enhanced production of chemokines. *Clinical & Experimental Rheumatology*.

[36] Álvarez-Soria M. A., Herrero-Beaumont G., Moreno-Rubio J. (2008). Long-term NSAID treatment directly decreases COX-2 and mPGES-1 production in the articular cartilage of patients with osteoarthritis. *Osteoarthritis and Cartilage*.

[37] Álvarez-Soria M. A., Largo R., Santillana J. (2006). Long term NSAID treatment inhibits COX-2 synthesis in the knee synovial membrane of patients with osteoarthritis: differential proinflammatory cytokine profile between celecoxib and aceclofenac. *Annals of the Rheumatic Diseases*.

[38] de Boer T. N., Huisman A. M., Polak A. A. (2009). The chondroprotective effect of selective COX-2 inhibition in osteoarthritis: *ex vivo* evaluation of human cartilage tissue after *in vivo* treatment. *Osteoarthritis and Cartilage*.

[39] Raynauld J.-P., Martel-Pelletier J., Beaulieu A. (2010). An open-label pilot study evaluating by magnetic resonance imaging the potential for a disease-modifying effect of celecoxib compared to a modelized historical control cohort in the treatment of knee osteoarthritis. *Seminars in Arthritis and Rheumatism*.

[40] Lewis J. S., Hembree W. C., Furman B. D. (2011). Acute joint pathology and synovial inflammation is associated with increased intra-articular fracture severity in the mouse knee. *Osteoarthritis and Cartilage*.

[41] Swärd P., Frobell R., Englund M., Roos H., Struglics A. (2012). Cartilage and bone markers and inflammatory cytokines are increased in synovial fluid in the acute phase of knee injury (hemarthrosis)—a cross-sectional analysis. *Osteoarthritis and Cartilage*.

[42] Bigoni M., Sacerdote P., Turati M. (2013). Acute and late changes in intraarticular cytokine levels following anterior cruciate ligament injury. *Journal of Orthopaedic Research*.

[43] Furman B. D., Mangiapani D. S., Zeitler E. (2014). Targeting pro-inflammatory cytokines following joint injury: acute intra-articular inhibition of interleukin-1 following knee injury prevents post-traumatic arthritis. *Arthritis Research & Therapy*.

[44] Lewis J. S., Furman B. D., Zeitler E. (2013). Genetic and cellular evidence of decreased inflammation associated with reduced incidence of posttraumatic arthritis in MRL/MpJ mice. *Arthritis and Rheumatism*.

[45] Holland T. A., Mikos A. G. (2003). Advances in drug delivery for articular cartilage. *Journal of Controlled Release*.

[46] Nasr M. (2010). In vitro and in vivo evaluation of proniosomes containing celecoxib for oral administration. *AAPS PharmSciTech*.

