# Supplementary material for: Study of Osteoarthritis Treatment with Anti-Inflammatory Drugs: Cyclooxygenase-2 Inhibitor and Steroids
Source: Biomed Res Int. 2015 Apr 27;2015:595273. doi: 10.1155/2015/595273 (PMC4427003; doi:10.1155/2015/595273)
Supplement: Supplementary file 1 — S. Figure 1. The effect of TNF-a concentration on MMP-1 and MMP-13 gene expression in pig chondrocytes. S. Figure 2. Effect of COX-2 inhibitor on MMP-1 and MMP-13 gene expression. S. Figure 3. Cell Viability in 0.1% DMSO. [file 595273.f1.pptx]

## Slide 1
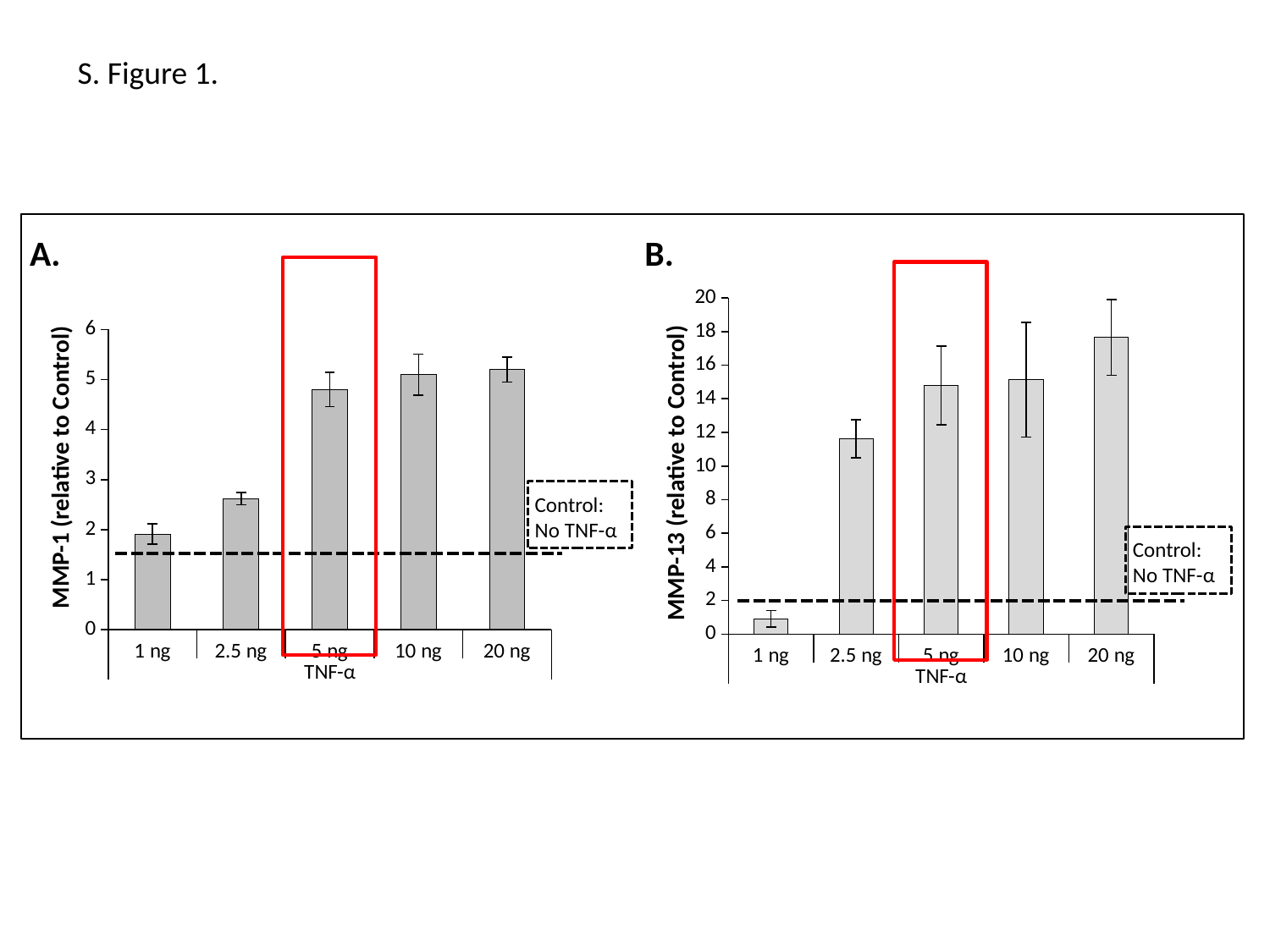

S. Figure 1.
A.
B.
### Chart
| Category | |
|---|---|
| 1 ng | 0.91 |
| 2.5 ng | 11.62 |
| 5 ng | 14.79 |
| 10 ng | 15.13 |
| 20 ng | 17.66 |
### Chart
| Category | |
|---|---|
| 1 ng | 1.91 |
| 2.5 ng | 2.62 |
| 5 ng | 4.8 |
| 10 ng | 5.1 |
| 20 ng | 5.2 |MMP-13 (relative to Control)
MMP-1 (relative to Control)
Control:
No TNF-α
Control:
No TNF-α
