# Supplementary material for: Study of Osteoarthritis Treatment with Anti-Inflammatory Drugs: Cyclooxygenase-2 Inhibitor and Steroids
Source: Biomed Res Int. 2015 Apr 27;2015:595273. doi: 10.1155/2015/595273 (PMC4427003; doi:10.1155/2015/595273)
Supplement: Supplementary file 2 [file 595273.f2.pptx]

## Slide 1
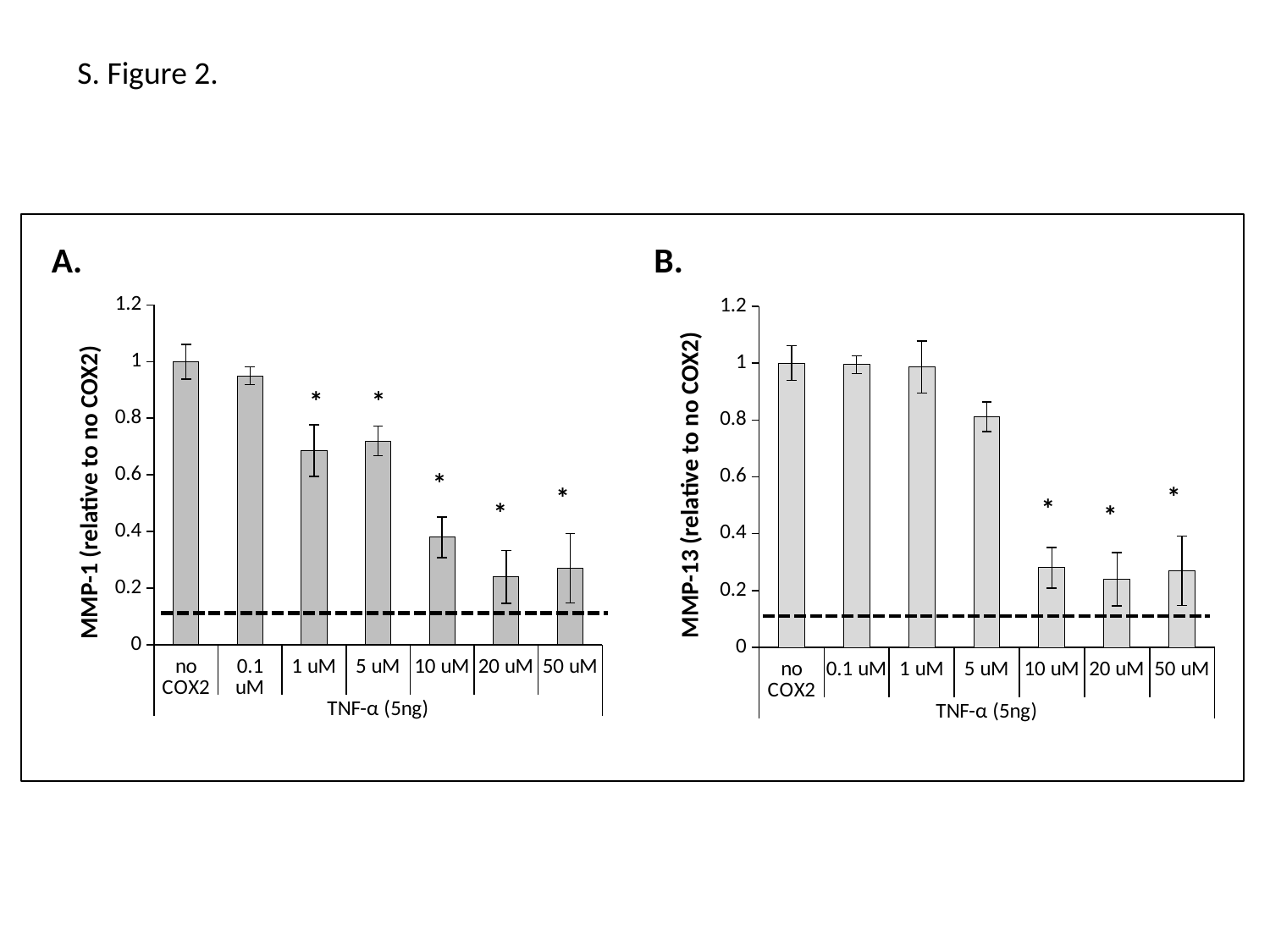

S. Figure 2.
A.
B.
### Chart
| Category | |
|---|---|
| no COX2 | 1.0 |
| 0.1 uM | 0.95 |
| 1 uM | 0.686 |
| 5 uM | 0.72 |
| 10 uM | 0.38 |
| 20 uM | 0.24 |
| 50 uM | 0.27 |
### Chart
| Category | |
|---|---|
| no COX2 | 1.0 |
| 0.1 uM | 0.995 |
| 1 uM | 0.986 |
| 5 uM | 0.812 |
| 10 uM | 0.28 |
| 20 uM | 0.24 |
| 50 uM | 0.27 |*
*
MMP-13 (relative to no COX2)
MMP-1 (relative to no COX2)
*
*
*
*
*
*
