# Supplementary figures and images for: Study of Osteoarthritis Treatment with Anti-Inflammatory Drugs: Cyclooxygenase-2 Inhibitor and Steroids
Source: Biomed Res Int. 2015 Apr 27;2015:595273. doi: 10.1155/2015/595273 (PMC4427003; doi:10.1155/2015/595273)

## Slide 1
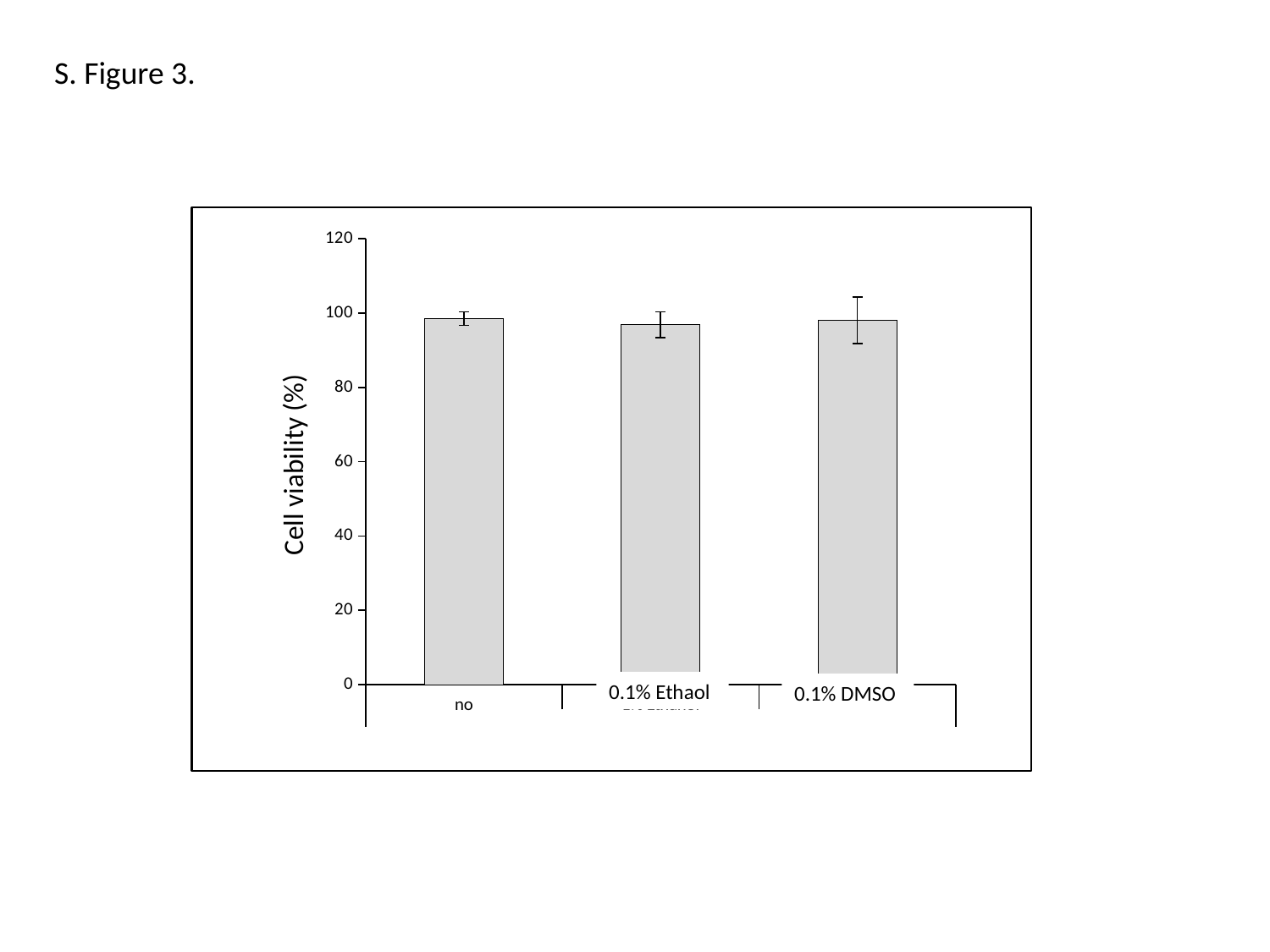

S. Figure 3.
### Chart
| Category | |
|---|---|
| no | 98.5 |
| 1% Ethanol | 96.9 |
| 1% DMSO | 98.1 |Cell viability (%)
0.1% Ethaol
0.1% DMSO

Supplement: Supplementary file 3 [file 595273.f3.pptx]
